# Repurposing ketamine to treat cocaine use disorder: integration of artificial intelligence-based prediction, expert evaluation, clinical corroboration and mechanism of action analyses

**DOI:** 10.1111/add.16168

**Published:** 2023-02-23

**Authors:** Zhenxiang Gao, T. John Winhusen, Maria Gorenflo, Udi E. Ghitza, Pamela B. Davis, David C. Kaelber, Rong Xu

**Affiliations:** 1 Center for Artificial Intelligence in Drug Discovery, School of Medicine, Case Western Reserve University, Cleveland, OH, USA; 2 Center for Addiction Research, University of Cincinnati College of Medicine, Cincinnati, OH, USA; 3 Cleveland Clinic Lerner College of Medicine, Case Western Reserve University, Cleveland, OH, USA; 4 Center for the Clinical Trials Network (CCTN), National Institute on Drug Abuse (NIDA), National Institutes of Health (NIH), Bethesda, MD, USA; 5 Center for Community Health Integration, School of Medicine, Case Western Reserve University, Cleveland, OH, USA; 6 Center for Clinical Informatics Research and Education, The Metro Health System, Cleveland, OH, USA

**Keywords:** Artificial intelligence, clinical corroboration, cocaine use disorder, drug repurposing, expert evaluation, ketamine, mechanism of action analyses

## Abstract

**Background and aims:**

Cocaine use disorder (CUD) is a significant public health issue for which there is no Food and Drug Administration (FDA) approved medication. Drug repurposing looks for new cost-effective uses of approved drugs. This study presents an integrated strategy to identify repurposed FDA-approved drugs for CUD treatment.

**Design:**

Our drug repurposing strategy combines artificial intelligence (AI)-based drug prediction, expert panel review, clinical corroboration and mechanisms of action analysis being implemented in the National Drug Abuse Treatment Clinical Trials Network (CTN). Based on AI-based prediction and expert knowledge, ketamine was ranked as the top candidate for clinical corroboration via electronic health record (EHR) evaluation of CUD patient cohorts prescribed ketamine for anesthesia or depression compared with matched controls who received non-ketamine anesthesia or antidepressants/midazolam. Genetic and pathway enrichment analyses were performed to understand ketamine’s potential mechanisms of action in the context of CUD.

**Setting:**

The study utilized TriNetX to access EHRs from more than 90 million patients world-wide. Genetic- and functional-level analyses used DisGeNet, Search Tool for Interactions of Chemicals and Kyoto Encyclopedia of Genes and Genomes databases.

**Participants:**

A total of 7742 CUD patients who received anesthesia (3871 ketamine-exposed and 3871 anesthetic-controlled) and 7910 CUD patients with depression (3955 ketamine-exposed and 3955 antidepressant-controlled) were identified after propensity score-matching.

**Measurements:**

EHR analysis outcome was a CUD remission diagnosis within 1 year of drug prescription.

**Findings:**

Patients with CUD prescribed ketamine for anesthesia displayed a significantly higher rate of CUD remission compared with matched individuals prescribed other anesthetics [hazard ratio (HR) = 1.98, 95% confidence interval (CI) = 1.42–2.78]. Similarly, CUD patients prescribed ketamine for depression evidenced a significantly higher CUD remission ratio compared with matched patients prescribed antidepressants or midazolam (HR = 4.39, 95% CI = 2.89–6.68). The mechanism of action analysis revealed that ketamine directly targets multiple CUD-associated genes (BDNF, CNR1, DRD2, GABRA2, GABRB3, GAD1, OPRK1, OPRM1, SLC6A3, SLC6A4) and pathways implicated in neuroactive ligand-receptor interaction, cAMP signaling and cocaine abuse/dependence.

**Conclusions:**

Ketamine appears to be a potential repurposed drug for treatment of cocaine use disorder.

## INTRODUCTION

As of 2020, more than 1.3 million adults suffer from cocaine use disorder (CUD) in the United States [1]. CUD has been a significant public health problem associated with elevated morbidity and mortality [2, 3]. However, there is no Food and Drug Administration (FDA)-approved medication to treat CUD [4, 5]. The standard treatment course for CUD involves psychosocial counseling and support, which may have limited efficacy or implementation in practice on its own. The discovery of medical treatments to supplement behavioral therapies for treatment of CUD is a high public health priority [6].

The traditional drug discovery process for medication development is lengthy and costly [7, 8]. Drug repurposing is a technique that discovers new indications for approved drugs in cost-effective ways [9–11]. There are some successful examples of drug repurposing, such as the use of rituximab for rheumatoid arthritis [12], metformin for various cancers [13, 14] and topiramate for obesity [15]. With the explosion of large-scale biomedical databases, numerous computational approaches have been developed to systematically analyze biological data to identify new treatments for various diseases [16, 17]. These approaches can be broadly categorized into network-based models [18, 19], structure-based approaches [20, 21] and artificial intelligence (AI)-based approaches [22, 23]. While these methods generate many repurposing signals, there have also been many repurposing drug candidates that have failed in clinical trial testing due to lack of efficacy [11, 24]. Thus, candidate drugs should undergo additional vetting prior to being tested in a clinical trial, which can be accomplished by integrating data from electronic health records (EHR) and input from experts in pharmacology and biomedical research [25].

The National Drug Abuse Treatment Clinical Trials Network (CTN) is conducting the ‘Drug Repurposing for cocaine use disorder (CUD)’ study using a combined strategy of AI-based prediction and retrospective clinical corroboration (CTN-0114). In this study, we developed a drug repurposing strategy that combined AI-based drug prediction, expert panel review, clinical corroboration through EHR-based testing and data-driven mechanisms of action analysis. Our drug repurposing strategy is highly generalizable and dynamic, and can be used for drug discovery for other substance use disorders. The present paper describes the outcome for a top candidate that completed the process: ketamine, a small synthetic organic molecule used clinically as an anesthetic and as a depression treatment.

## MATERIAL AND METHODS

**FIGURE 1:**
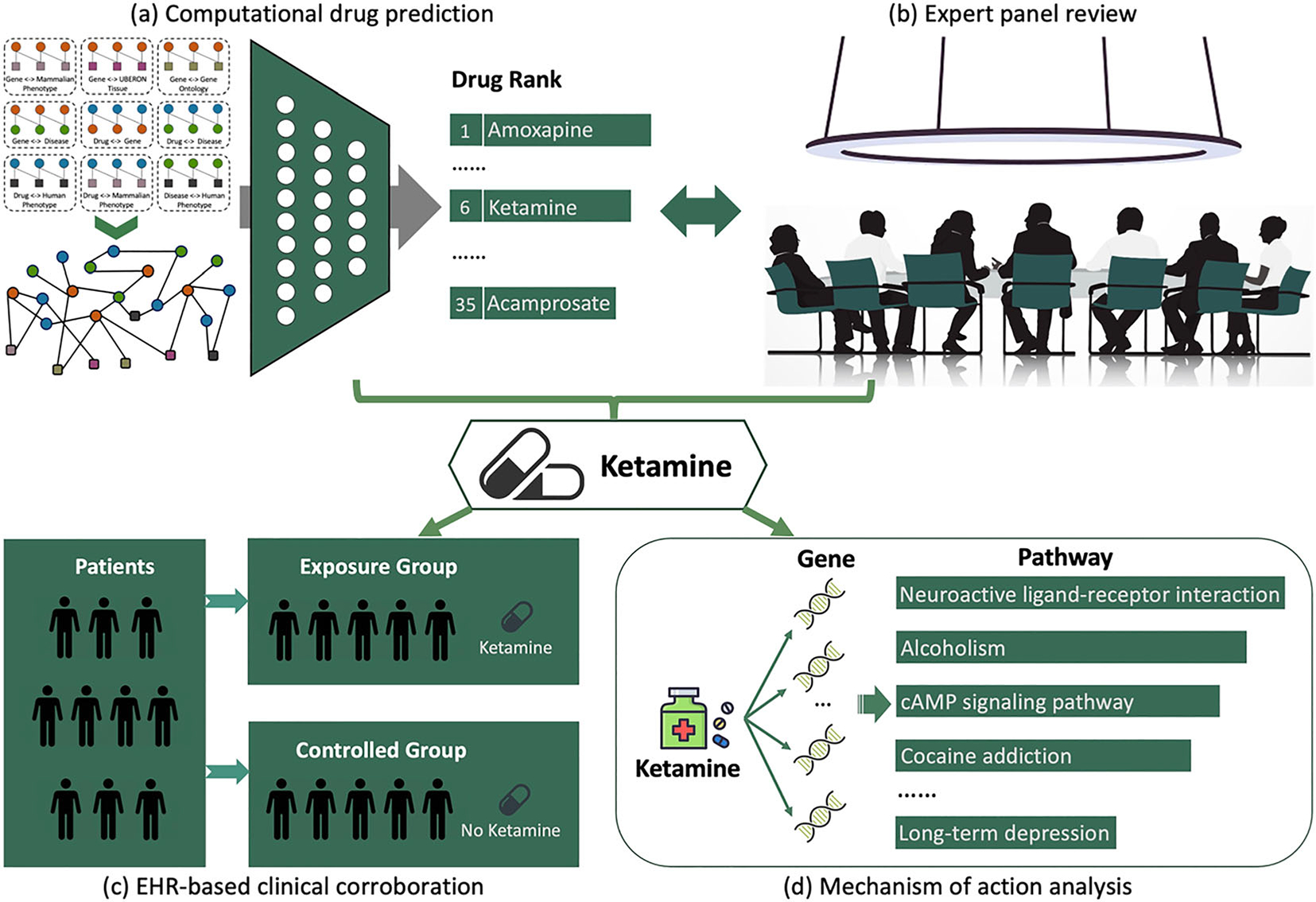
The pipeline of the drug repurposing strategy for cocaine use disorder. (a) Our knowledge graph-based drug discovery system modeled multi-type interactions from various biomedical databases to rank candidate drugs for cocaine use disorder (CUD) treatment. (b) The CTN-0114 advisory committee reviewed the top-ranked drugs and selected ketamine for further clinical evaluation. (c) Electronic health record (EHR)-based analysis provided clinical corroborations of ketamine for CUD treatment. (d) The genetic and functional-level analyses showed that ketamine directly targets multiple CUD genes and pathways.

Our study consisted of the following steps (Figure 1). (1) We identified potential drug candidates from 1430 FDA-approved drugs using KG-Predict, a knowledge-driven AI-based system that we previously developed for general purpose drug discovery and disease gene prediction [26, 27]. (2) The CTN-0114 advisory committee reviewed the top 35 drug candidates ranked by KG-Predict. (3) We performed retrospectives control studies to evaluate the potential efficacy of a single drug (ketamine) for CUD treatment using EHR data from 90 million patients. (4) We performed genetic analysis and Kyoto Encyclopedia of Genes and Genomes (KEGG) pathway enrichment analysis [28] to understand the potential mechanisms of action of ketamine in the context of CUD.

### Knowledge graph-based drug discovery system

Knowledge graph, a powerful AI technology to abstract, organize and integrate knowledge extracted from multiple data sources, has become an emerging technology for biomedical discovery. We constructed a knowledge graph-based prediction system, KG-Predict, that prioritizes candidate drugs for a given input disease by modeling the interconnections between drugs, genes, diseases and phenotypical annotations from publicly available phenome-level databases, genome-level databases and text-mined knowledge bases [26, 27]. In KG-Predict, the associations between drugs and their corresponding phenotypes were obtained from the Phenomebrowser [29] database. The associations between genes and their functions were obtained from Gene Ontology Annotation (GOA) [30], Mouse Genome Informatics (MGI) [31] and Genotype-Tissue Expression (GTEx) [32] databases. The associations between diseases and phenotype ontologies were obtained from the human phenotype ontology (HPO) database [33]. The associations between drugs and genes were obtained from the DrugBank database [34]. We extracted disease–gene interactions from the MGI database. The drug–disease interactions were mined by natural language processing (NLP) techniques from records of patients in FAERS, FDA drug labels, MED-LINE abstracts and clinical trial studies [35, 36]. The knowledge graph in KG-Predict was composed of seven types of entities (e.g. drugs, genes, diseases, phenotypical annotations) linked by nine types of semantic relations (e.g. drug–target–gene, gene–associate–GOA). More details are provided in Supporting information, Table S1 in the supporting information.

In this study, the input to KG-Predict is a list of CUD-associated genes. The output is a list of candidate drugs prioritized based on their genetic, genomic and phenotypical relevance to CUD. To collect CUD-associated genes, we first obtained 383 genes that are associated with cocaine-related diseases (cocaine dependence, cocaine abuse and cocaine-related disorder) from DisGeNet [37], a discovery platform with one of the largest publicly available collections of genes and variants associated with human diseases. We used the median score of 0.5 as the threshold. At a cut-off score of 0.5, 19 genes were associated with cocaine-related diseases. We also obtained three CUD-associated genes from the published literature [38–40]. The CUD-associated gene list included DRD3, GABRA2, CAMK4, MECP2, OPRK1, COMT, CREB1, CARTPT, CRH, CNR1, CRHR1, OPRM1, SLC6A4, NPY, PDYN, DRD2, HTR1B, SLC6A3, EGR1, GAD1, GABRB3 and BDNF.

### Expert panel review from pharmacology or biomedical research fields

The names of the CTN-0114 advisory committee members are listed in the Acknowledgements section. The committee includes experts in data science, addiction psychiatry and addiction treatment. All seven members are seasoned CTN investigators, with the majority boasting more than 20 years’ experience conducting addiction trials, including multiple trials testing medications for the treatment of CUD. The role of the advisory committee was to provide feedback on the potential clinical utility of the identified candidates with the investigative team making the decision about which candidates to submit to EHR analysis. A REDCap [41] survey was created to obtain input from each advisory committee member. For each drug, the advisory committee member was asked whether the candidate should be included in the EHR analysis taking into account: ‘(1) existing pre-clinical and clinical trials evidence, (2) likely challenges with clinical utilization, (3) the potential to address co-occurring substance use and/or to phenotypes which patients frequently report as being prominent barriers to their recovery and (4) on-going (or soon to start investigations)’.

### EHR-based large-scale clinical corroborations for CUD remission using the expert panel-selected medication (ketamine)

#### Study setting

This study utilized TriNetX [42], a federated health research network, to access EHRs from approximately 90 million unique patients across the United States. TriNetX Analytics provides web-based secure access to patient EHR data from hospitals, primary care clinics and specialty treatment providers, covering demographics, diagnoses, procedures, medications, laboratory testing, vital signs and genomic information. The platform features built-in functions that allow for cohort selection, matching incidence and prevalence analysis and comparison of characteristics and outcomes between matched cohorts. TriNetX only uses aggregate counts and statistical summaries of deidentified patients, so no protected health information or personal data are available to users. Consequently, the MetroHealth System, Cleveland Ohio, Institutional Review Board has determined that any research using TriNetX is not Human Subject Research and is therefore exempt from review. We previously used the TriNetX Analytics network platform to perform large-scale cohort studies in patients with CUD and other diseases [43–54], including evaluating potential clinical efficacy of repurposed drugs in real-world populations [55, 56].

Ketamine exposure information was obtained from pharmacy data in TriNetX, including the drug name, issue dates of prescription and order records. Ketamine was coded as National Drug Code (NDC, the FDA drug identification system) 6130. In addition, we collected drug information for 10 widely utilized anesthetics for analysis as comparison drugs: propofol (NDC 8782), methohexital (NDC 6847), thiopental (NDC 10493), isoflurane (NDC 6026), desflurane (NDC 27340), etomidate (NDC 4177), sevoflurane (NDC 36453), fospropofol (NDC 828682), enflurane (NDC 3920) and midazolam (NDC 6960). Midazolam and the class of antidepressants (NDC CN600) were also used as comparison drugs in a subsequent analysis.

The status of CUD was based on the International Classification of Diseases 10th revision (ICD-10) diagnosis code for ‘cocaine-related disorder’ (ICD F14). The status of anesthesia was based on the Current Procedural Terminology (CPT) relevant codes for ‘Anesthesia’ (CPT 1002796) and the ICD-10 diagnosis code for ‘Anesthesia of skin’ (ICD R20.0). The status of depression was based on the ICD-10 diagnosis code for ‘depressive episode’ (ICD F32) and ‘major depressive disorder, recurrent’ (ICD F33). The outcome ‘remission from CUD’ was based on the diagnosis of ‘cocaine abuse in remission’ (ICD F14.11) or ‘cocaine dependence in remission’ (ICD F14.21).

#### Study population

Using the TriNetX database, we identified a total of 379 409 patients diagnosed with CUD between January 2007 and June 2022. Among the population with CUD, 16 754 patients were prescribed ketamine.

The following studies were conducted:

1. We first investigated if patients with CUD who were prescribed ketamine for anesthesia have a higher CUD remission rate compared with patients prescribed other anesthetics. The exposure group was composed of patients who were prescribed ketamine within 1 year of their initial CUD diagnosis but who were never in remission from CUD prior to ketamine prescription. The control group included patients who were never prescribed ketamine but who were prescribed another anesthetic within 1 year of their initial CUD diagnosis; they had also never experienced remission from CUD prior to this anesthetic prescription.
2. We investigated if CUD patients with unipolar or major depression who were prescribed ketamine (75% of patients received a single prescription and 25% received two or more prescriptions) displayed higher CUD remission rates compared with individuals prescribed other drugs to treat depression, such as antidepressants or midazolam (midazolam was used as a control in the real-life clinical trials that have investigated ketamine administration for CUD [57–59]). The exposure group included CUD patients with depression who were prescribed ketamine within 1 year of their initial CUD diagnosis but who were never in remission from CUD before ketamine prescription. The control group included CUD patients with depression who were never prescribed ketamine but who were prescribed either midazolam or antidepressants within 1 year of their initial CUD diagnosis; they also never experienced remission from CUD before their midazolam or antidepressant prescription.
3. As there is a 25% overlap between CUD patients who received anesthesia and those with unipolar or major depression, we performed additional analyses to examine potential effects of ketamine on CUD remission in two non-overlapping cohorts of CUD patients. (a) The exposure group included CUD patients who were never diagnosed with depression but prescribed ketamine for anesthesia. The control group included patients who were never prescribed ketamine and never diagnosed with depression but who were prescribed another anesthetic for anesthesia within 1 year of their initial CUD diagnosis; they had also never experienced remission from CUD prior to this anesthetic prescription. (b) The exposure group was composed of CUD patients who never received anesthesia and were prescribed ketamine for depression. The control group included CUD patients with depression who were never prescribed ketamine and never received anesthesia, but who were prescribed either midazolam or antidepressants within 1 year of their initial CUD diagnosis; they also never experienced remission from CUD before their midazolam or antidepressant prescription.

**FIGURE 2:**
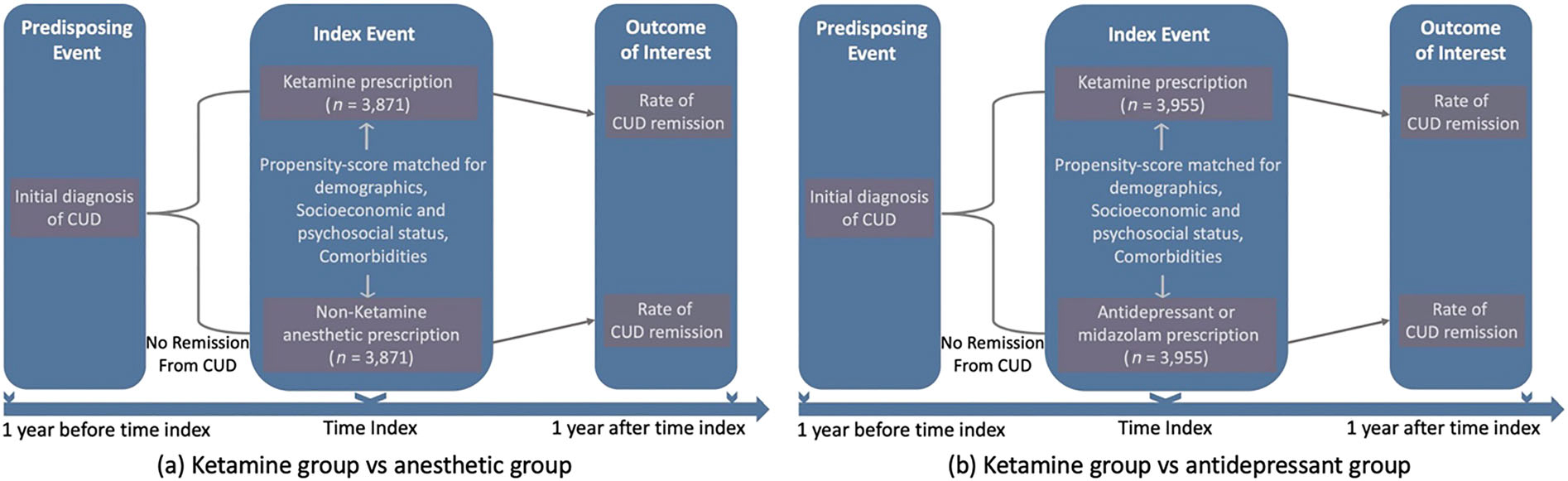
Flow-charts of retrospective case–control cohort design.

The index event for each study was the date of drug prescription (either ketamine, other anesthetics or antidepressant/midazolam). Patients who experienced remission from CUD prior to drug prescription or those whose initial CUD diagnosis was more than 1 year before drug prescription was excluded (Figure 2).

### Propensity score-matching

To balance the cohorts, the TriNetX built-in propensity score-matching function was used, which involves 1:1 matching using a nearest-neighbor greedy matching algorithm with a caliper of 0.1 standardized mean differences (SMD) to account for potential confounding variables [45, 46, 60]. The purpose of propensity score-matching was to render the exposure and control groups more comparable by accounting for and reducing the bias of covariates that may act as confounding variables.

The list of covariates, as well as their standardized name codes and data types used in TriNetX, is described in the Supporting information, Table S2. These covariates included demographics (age, gender, race/ethnicity): socio-economic and psychosocial status (ICD Z55–Z65 for ‘Persons with potential health hazards related to socio-economic and psychosocial circumstances’), which denotes problems with employment, housing, education and economic circumstances; mental and behavioral disorders due to psychoactive substance use (ICD F10–F19), encompassing opioid-related disorders, alcohol-related disorders, cannabis-related disorders, nicotine dependence and hallucinogen-related disorders; schizophrenia and other non-mood psychotic disorders (ICD F20–F29); mood disorders (ICD F30–F39), including depression and bipolar disorder as well as anxiety, dissociative, stress-related, somatoform and other non-psychotic mental disorders (ICD F40–F48); attention-deficit/hyperactivity disorder (ICD F90); conduct disorders (ICD F91); and relevant life-time comorbidities (hypertension (ICD I10-I16), heart disease (ICD I20–I25, I30–I5A), cerebrovascular disease (ICD I60–I69) and acute kidney disease (ICD N17). These covariates are all implicated in the development and severity of CUD [61–65].

### Statistical analyses

Comparisons between cohorts were made using Cox’s proportional hazards model (a built-in function in TriNetX). The diagnosis of remission from CUD within 1 year of drug prescription was the outcome of interest. Hazard ratios (HR) and 95% confidence intervals (CI) were calculated to compare the outcome of interest between cohorts based on comparison of time to event rates. The proportional hazard assumption was tested using the generalized Schoenfeld approach [66]. We examined the overall HR of CUD remission in patients prescribed ketamine compared with matched patients prescribed other anesthetics or antidepressants/midazolam (Figure 2). We also stratified the analysis by gender and race. The analysis was not pre-registered, and therefore the results should be considered exploratory rather than confirmatory.

### Genetic- and functional-level analyses of ketamine in the context of CUD

#### Genetic-level analysis

We used the STITCH (Search Tool for Interactions of Chemicals) database to obtain the protein-coding genes associated with ketamine. STITCH contains data on the interactions between 500 000 small molecules and 9.6 million proteins from 2031 organisms [67]. The scores of associations between chemicals and protein-encoding genes range from 0.001 to 0.999. The median score of 0.5 is used as the cut-off value. We then determined how many genes were associated with both ketamine and CUD.

#### Functional-level analysis

We identified the CUD-associated pathways that ketamine directly targets to more clearly understand the potential causal relationship between ketamine use and CUD remission. Genetic pathways for each CUD gene (listed in the Results section) were first obtained from the KEGG database, which stores high-level functions and utilities of biological systems [28]. For each pathway, we assessed its probability of being associated with the given set of CUD-associated genes at a significance threshold of *P* < 0.01. Genetic targets of ketamine were also obtained from the STITCH database. Genetic pathways that are significantly associated with ketamine-associated genes were identified from the KEGG database [28]. For each pathway, *P*-values ≥ 0.01 were discarded. Finally, we intersected ketamine-associated pathways with CUD-associated pathways to determine which pathways are implicated in both CUD and ketamine.

## RESULTS

### Top repurposed candidate drugs for treating CUD identified by the knowledge graph-based drug discovery system

The top 10 drugs ranked by our knowledge graph-based drug discovery system are listed in Table 1. Three of these (aripiprazole, ketamine and quetiapine) have been evaluated as CUD treatments in clinical trials (https://clinicaltrials.gov/), and clozapine was implicated as CUD treatments in published literature [68]. All 10 drugs are approved for treating either depression or schizophrenia, which are frequently comorbid with CUD [61].

### Expert panel ratings

Based on the enrichment of potential anti-CUD drugs from clinical trials (https://clinicaltrials.gov/) and evidence from PubMed, the top 35 (2.5% of 1430) FDA-approved drugs identified by our knowledge graph-based drug discovery system were provided to the advisory committee (Supporting information, Table S3). The advisory committee ratings of the 35 candidate drugs are provided in Supporting information, Table S4. Ketamine ranked sixth among top 35 candidate drugs and was the only drug candidate that all advisory committee members recommended for inclusion in the EHR analysis; based on this consensus, the investigative team decided to complete the EHR-based evaluation of ketamine.

### EHR-based analysis evaluating ketamine for CUD treatment

#### Ketamine is associated with significantly greater remission from CUD in patients prescribed ketamine as an anesthetic

**FIGURE 3:**
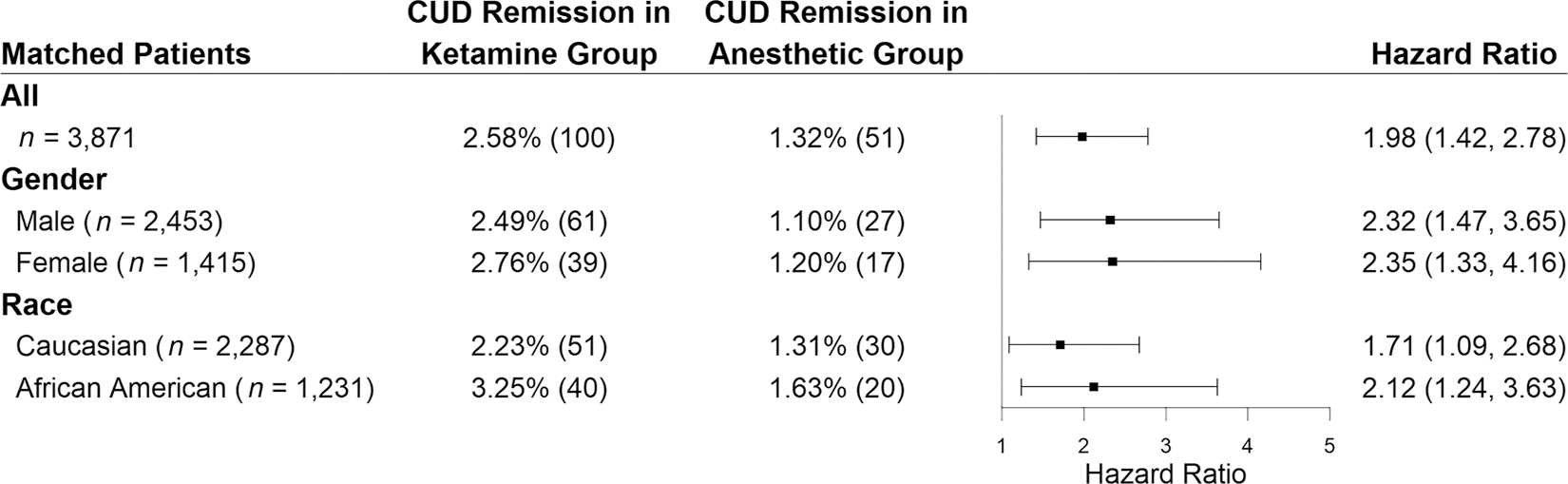
Hazard ratios for remission from cocaine use disorder (CUD) in patients prescribed with ketamine compared with propensity score-matched patients prescribed with other anesthetics.

The cohort of CUD patients prescribed ketamine differed from the unmatched sample prescribed other anesthetics in age, race, comorbidities and socio-economic and psychosocial status. As can be seen in Table 2, the matching procedure created comparable matched groups of 7742 CUD patients who received anesthesia (3871 ketamine-exposed and 3871 anesthetic-controlled), with no significant differences on patient characteristics. Patients prescribed ketamine displayed a significantly higher ratio of CUD remission compared with propensity score-matched individuals prescribed other anesthetics without ketamine (HR = 1.98, 95% CI = 1.42–2.78) (Figure 3). Similar findings were observed in patients stratified by gender with an HR of 2.32 (95% CI = 1.47–3.65) for women and 2.35 (95% CI = 1.33–4.16) for men and in populations stratified by race with an HR of 1.71 (95% CI = 1.09–2.68) for White and 2.12 (95% CI = 1.24–3.63) for Black patients. In addition, we found that no significant gender or race disparities for CUD remission in patients taking ketamine (Supporting information, Figure S1).

#### Ketamine is associated with significantly greater remission from CUD in patients prescribed ketamine as an antidepressant

**FIGURE 4:**
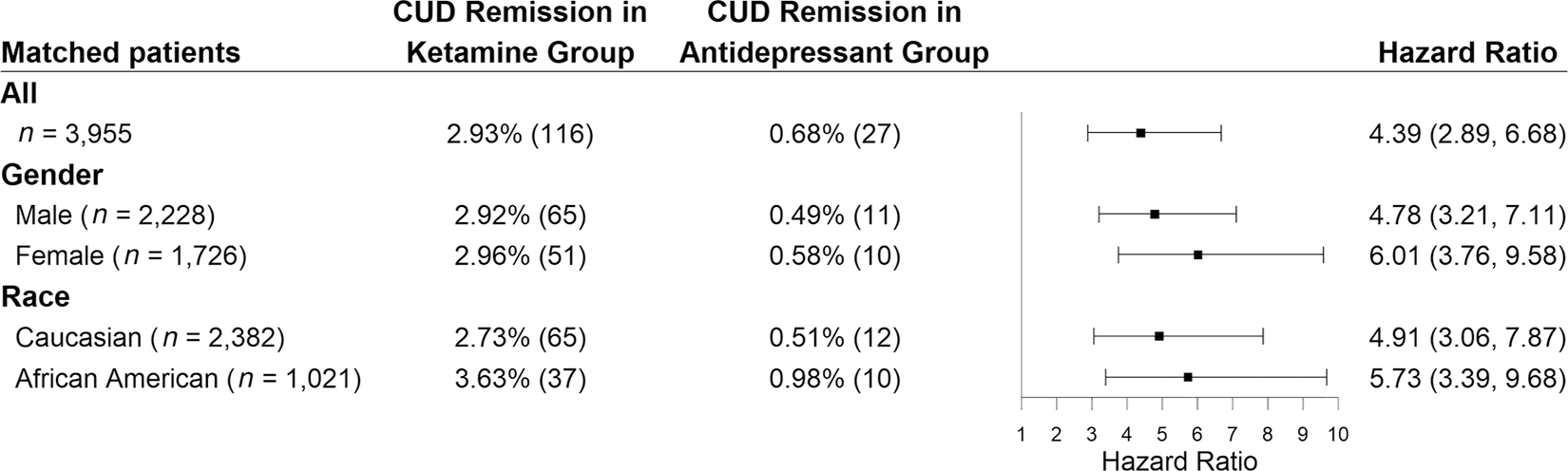
Hazard ratios for remission from cocaine use disorder (CUD) in patients with depression prescribed with ketamine compared with propensity score-matched patients prescribed with other antidepressants or midazolam.

The characteristics of CUD patients prescribed ketamine versus antidepressants or midazolam are shown in Table 3. As can be seen in Table 3, the matching procedure created comparable matched groups of 7910 CUD patients with depression (3955 ketamine-exposed and 3955 antidepressant-controlled), with no significant differences on patient characteristics. Patients prescribed ketamine displayed a significantly higher CUD remission ratio compared with patients in matched antidepressant groups with an HR of 4.39 (95% CI = 2.89–6.68). Similar findings were observed in population stratified by gender and race (Figure 4). There were also no significant gender or race disparities for CUD remission in patients taking ketamine (Supporting information, Figure S2).

#### Ketamine is associated with significantly greater remission from CUD in two non-overlapping cohorts of patients

**FIGURE 5:**
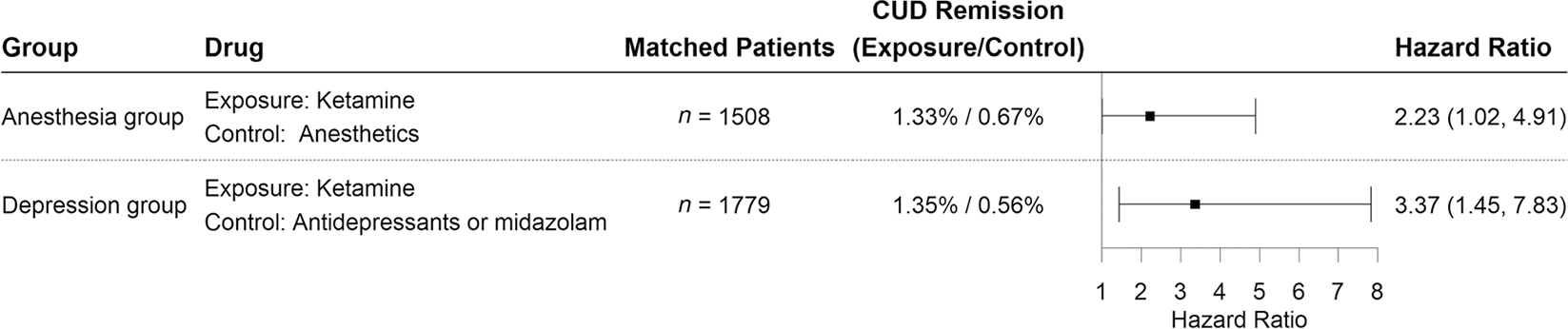
Clinical efficacy of ketamine for cocaine use disorder (CUD) remission in two non-overlapping cohorts of CUD patients.

To further clarify the impact of overlap between the anesthesia and depression patient samples, we stratified the analysis by dividing CUD patients into two non-overlapping cohorts (Figure 5). The patient characteristics were provided in Supporting information, Tables S5 and S6. For the anesthesia group, we identified an EHR cohort of CUD patients who were never diagnosed with depression and prescribed ketamine for anesthesia. The result showed that patients with CUD who were administered ketamine displayed higher rates of remission from CUD compared with matched individuals prescribed other anesthetics (HR = 2.23, 95% CI = 1.02–4.91). For the depression group, we identified an EHR cohort of CUD patients who never received anesthesia and were prescribed ketamine for depression. The result similarly displayed higher rates of remission from CUD compared with matched individuals prescribed other antidepressants or midazolam (HR = 3.37, 95% CI = 1.45–7.83).

### Ketamine is involved in CUD at the genetic and functional levels

We analyzed how ketamine relates to CUD at the genetic and functional levels. At the genetic-level, we obtained 154 genes associated with ketamine, among which 10 genes are implicated in CUD, including BDNF, CNR1, DRD2, GABRA2, GABRB3, GAD1, OPRK1, OPRM1, SLC6A3 and SLC6A4. The complete list of CUD- and ketamine-associated genes is provided in Supporting information, Table S7.

We then analyzed the functional relationship between ketamine and CUD. We identified 13 genetic pathways that are significantly enriched for the 24 CUD-associated genes from the KEGG database. The most enriched CUD pathways involve neuroactive ligand–receptor interaction, alcoholism, cAMP signaling and cocaine addiction (see Supporting information, Table S8 for the full list). A total of 78 significantly enriched pathways for the 154 ketamine-associated genes were obtained from the KEGG database. We identified significantly enriched pathways for ketamine and the shared pathways between ketamine and CUD. As shown in Table 4, 12 pathways were significantly enriched for both CUD and ketamine (92% of 13 CUD pathways). Several pathways that were shared by CUD- and ketamine-associated genes, but not significantly enriched for either CUD or ketamine, included apelin signaling pathway and synaptic vesicle cycle.

## DISCUSSION

In this study, we implemented a drug repurposing strategy for CUD treatment by combining AI-based drug prediction, expert panel review, large-scale clinical corroboration based on EHRs and data-driven mechanism of action analysis. The AI system generated a list of candidates based on a large amount of genetic, genomic and phenotypical data from both human and mouse models. Experts refined the list of candidates based on probable clinical utility and unanimously selected ketamine for further EHR and mechanism of action analyses, the results of which suggest that ketamine has the potential to improve remission rates in patients with CUD.

Ketamine is a rapid-acting general anesthetic that is typically administered as a single injection [69]. We identified an EHR cohort of CUD patients prescribed ketamine for anesthesia and found that patients with CUD who were administered ketamine displayed higher rates of remission from CUD compared with individuals prescribed other anesthetics. Another indication for ketamine is treatment-resistant depression, for which subanesthetic doses of ketamine can trigger rapid antidepressant-like effects [70, 71]. An HER-identified cohort of CUD patients who were prescribed ketamine for depression similarly displayed higher rates of remission from CUD compared with individuals prescribed other antidepressants or midazolam. While the absolute rates of remission were quite modest (1–3% in ketamine-treated patients), these data nevertheless provide a convincing signal for potential clinical efficacy in CUD.

The present results, which suggest a role for ketamine in the treatment of CUD, are consistent with several clinical trials evaluating ketamine in patients with CUD [57–59, 72]. These trials enrolled between eight and 50 individuals with CUD, utilizing either lorazepam (an anti-anxiety medication) or midazolam (a benzodiazepine) for the control group. These studies revealed that, compared with the control group, CUD patients administered ketamine experienced a significant reduction in cocaine craving [57, 59] and self-administration [58] shortly after taking ketamine and were three times as likely to remain abstinent from cocaine use during the last 2 weeks of a 5-week trial [72]. The combined results from these trials point to ketamine’s potential to treat CUD more effectively than other psychiatric medications. The trials conducted to date have included small homogenous samples limiting the generalizability of the findings [57–59, 72]. The results from the present study found that ketamine exposure was associated with CUD remission in patients with significant medical and psychiatric comorbidities and did not find differential effects by gender or race, suggesting that ketamine may show efficacy with more heterogeneous samples.

The mechanisms by which ketamine may impact cocaine use have not been delineated, although there is some evidence that the mystical-type experience that can arise from single-dose ketamine exposure might be important for ketamine’s impact [59, 73]. It is also important to consider the biochemical pathways that could explain ketamine’s potential as a treatment for CUD, considering the shared pathways we identified between ketamine and CUD. Ketamine targets a multitude of proteins in the brain implicated in the pathogenesis of addiction [74]. NMDA receptors are ketamine’s major target for its physiological effects; ketamine non-competitively antagonizes NMDA receptors by binding to them allosterically [75]. Cocaine affects NMDA receptors in various ways, although the specific biochemical linkages of this relationship have not been fully elucidated. Cocaine triggers changes to NMDA receptor subunits, DNA transcription downstream of NMDA receptor binding and re-assembly of protein complexes involved in the NMDA receptor pathway [76]. Cocaine also modulates cross-talk between NMDA and dopamine receptors, which is notable, as dopamine signaling in the mesocorticolimbic dopamine system is critical for the neurochemical development of addiction [77]. In addition to its interactions with NMDA receptors, ketamine influences the activity and expression of brain-derived neurotrophic factor (BDNF) [78], eukaryotic elongation factor 2 and mechanistic target of rapamycin (mTOR), which modulate synaptic plasticity, a process implicated in the pathogenesis of depression [79] and addiction [75]. Much work remains to be conducted to elucidate any causal relationship between ketamine use and CUD remission, both on the subjective patient and molecular levels. Future work should focus upon animal studies to define ketamine’s biochemical effects more concretely on the addicted brain, as well as larger-scale randomized clinical trials, to definitively demonstrate the benefits of ketamine for CUD patients.

We previously developed KG-Predict for general purpose drug discovery and disease genetic prediction [26, 27]. In the present project, we applied KG-Predict for CUD drug repurposing. Among top 35 AI-generated candidates reviewed by the expert panel, only one (ketamine) was unanimously selected for further evaluation, with most of the other candidates given a low rating based on probable adherence issues. Ketamine has previously been studied for treating CUD [57–59] and thus, in this case, the AI system was of limited utility in terms of generating truly novel candidates for treating CUD based on the top 35 candidates reviewed. However, the present study reinforces the importance of vetting AI-generated candidates with experts familiar with their clinical profiles. In addition, the selection of ketamine through the AI system lends further support to ketamine’s promise as a potential CUD treatment.

Our study has several limitations. First, information on drug usage duration, dosage and patient adherence is limited in the EHR database; for example, prescriptions may be obtained from outside providers that were not recorded in TriNetX. Due to these limitations, we could not evaluate how the duration, dosage and compliance of drug use affect CUD remission. Secondly, although widely used and accepted for observational studies on health-care utilization, drug utilization, epidemiology, risk factors and safety surveillance, the observational, retrospective nature of this study could introduce selection, information, testing and follow-up biases. Patient EHR data may suffer from underdiagnosis/overdiagnosis/misdiagnosis and do not include all possible confounding factors. One potential limitation may come from propensity score-matching. Although patient matching can make distributions of covariates more balanced to avoid many confounders, we could still miss some unobserved and unmeasured confounders due to the observational nature of the study design, which could increase data imbalance and bias. Thirdly, while our study used standard statistical analyses for cohort studies, it would benefit from additional sensitivity analysis as well as negative control analysis. Fourthly, although the TriNetX EHR database collects de-identified data from 90 million unique patients across the United States, patients in the EHR database represent people who had medical encounters with healthcare systems and do not necessarily represent the entire US population. While patients with CUD probably have medical encounters, the generalizability of the results from the TriNetX platform remains unknown and needs to be validated in other populations. Finally, the characterization of the genes and pathways involved in the pathogenesis, etiology and biochemistry of CUD is probably incomplete. In the future, newly identified CUD-related genes and pathways will improve the identification of potential drugs for CUD.

In conclusion, the results from our drug repurposing strategy combining AI-based drug prediction, expert panel review, clinical corroboration and mechanisms of action analysis support ketamine as a potential repurposed drug to treat CUD. These findings, taken together with findings from the small clinical trials that have tested ketamine for CUD, suggest that additional clinical trials are warranted.

## Supplementary Material

Supporting information

## Figures and Tables

**TABLE 1:** Top 10-ranked drug candidates associated with the input of CUD-related genes.

No.	Drug	Original indication	Evidence for CUD treatment

1	Amoxapine	Depression	
2	Aripiprazole	Schizophrenia	NCT00780702, NCT00276874
3	Loxapine	Schizophrenia	
4	Olanzapine	Schizophrenia	
5	Amitriptyline	Depression	
6	Ketamine	Anesthetic agent, depression	NCT01535937, NCT01790490
7	Clozapine	Schizophrenia	PMID10891628
8	Quetiapine	Schizophrenia	NCT00232336, NCT00631748
9	Ziprasidone	Schizophrenia	
10	Trimipramine	Depression	

*Note*: NCT = evidence from clinical trials (clinicaltrials.gov); PubMed reference number (PMID) = evidence from biomedical literature.

Abbreviation: CUD, cocaine use disorder.

**TABLE 2:** Characteristics of CUD patients in the ketamine treatment or other anesthetic treatment cohorts before and after propensity scorematching.

Characteristics	Before matching			After matching		
	Ketamine cohort	Anesthetic cohort	SMD	Ketamine cohort	Anesthetic cohort	SMD

Total no.	3872	13 868		3871	3871	
Age, mean (SD)	43.2 (13.1)	47.6 (13.1)	**0.33**	43.2 (13.1)	42.7 (13.5)	0.04
Sex, %						
Female	36.6	36.4	0.005	36.6	35.6	0.02
Male	63.4	63.6	0.005	63.4	64.4	0.02
Ethnicity, %						
Hispanic/Latinx	9.9	7.5	0.09	9.9	9.6	0.01
Not Hispanic/Latinx	81.4	81.6	0.005	81.4	82.1	0.02
Race, %						
African American/Black	31.8	44.5	**0.26**	31.8	32.5	0.01
White	59.1	47.7	**0.23**	59.1	58.7	0.01
Asian	0.4	0.2	0.03	0.4	0.3	0.02
Comorbidities, %						
Mental and behavioral disorders	88.9	84.6	**0.13**	88.9	87.8	0.04
Mood disorders	49.7	43.3	**0.13**	49.7	47.1	0.05
Anxiety	48.6	39.6	**0.18**	48.6	46.4	0.05
Schizophrenia	15.9	13.1	0.08	15.9	14.4	0.04
Hypertensive diseases	44.9	52.9	**0.16**	44.9	42.9	0.04
Ischemic heart diseases	22.8	26.5	0.09	22.8	20.6	0.05
Other forms of heart disease	48.8	44.6	0.09	48.8	46.1	0.05
Cerebrovascular diseases	19.5	20.3	0.02	19.5	17.8	0.04
Acute kidney failure	21.9	19.4	0.06	21.9	20.7	0.03
Antisocial personality disorder	1.3	0.8	0.04	1.3	1.2	0.01
Conduct disorder	9.9	6.8	**0.11**	9.9	9.3	0.02
ADHD	5.5	3.6	0.09	5.5	4.9	0.02
Socio-economic and psychosocial status	27.9	23.3	**0.11**	27.9	26.1	0.04

*Note:* Values shown in bold type are significant at SMD > 0.1.

Abbreviation: ADHD, attention-deficit/hyperactivity disorder; CUD, cocaine use disorder; SMD, standardized mean difference.

**TABLE 3:** Characteristics of CUD patients with depression receiving ketamine treatment or other antidepressant treatment before and after propensity score-matching.

Characteristics	Before matching			After matching		
	Ketamine cohort	Antidepressant cohort	SMD	Ketamine cohort	Antidepressant cohort	SMD

Total no.	3959	65 680		3955	3955	
Age, mean (SD)	42.5 (13.2)	42.6 (14.3)	0.003	42.5 (13.2)	43.1 (14.1)	0.04
Sex, %						
Female	43.7	45.4	0.03	43.6	43.2	0.009
Male	56.3	54.6	0.04	56.4	56.7	0.008
Ethnicity, %						
Hispanic/Latinx	8.7	6.8	0.07	8.6	7.9	0.02
Not Hispanic/ Latinx	76.1	72.7	0.08	76.1	78.3	0.05
Race, %						
African American/Black	25.8	32.1	**0.14**	25.8	26.2	0.007
White	60.2	56.1	0.08	60.2	60.7	0.01
Asian	0.5	0.6	0.02	0.5	0.4	0.01
Comorbidities, %						
Mental and behavioral disorders	90.6	81.5	**0.26**	90.6	90.8	0.006
Mood disorders	76.2	71%	**0.12**	76.2	77.2	0.02
Anxiety	62.3	49.1	**0.27**	62.3	61.7	0.01
Schizophrenia	18.3	16.1	0.06	18.3	16.4	0.05
Hypertensive diseases	45.8	35.5	**0.21**	45.8	47.9	0.04
Ischemic heart diseases	21.7	12.8	**0.24**	21.6	22.2	0.01
Other forms of heart disease	46.1	24.1	**0.47**	46.1	47.8	0.04
Cerebrovascular diseases	16.9	9.1	**0.23**	16.7	16.6	0.004
Acute kidney failure	23.3	10.1	**0.36**	23.2	22.6	0.01
Antisocial personality disorder	1.5	1.3	0.01	1.5	1.1	0.04
Conduct disorder	9.9	4.6	**0.21**	9.8	8.5	0.04
ADHD	7.5	6.3	0.05	7.5	5.9	0.06
Socio-economic and psychosocial status	31.6	21.2	**0.24**	31.5	29.3	0.05

*Note:* Values shown in bold type are significant at SMD > 0.1.

Abbreviation: ADHD, attention-deficit/hyperactivity disorder; CUD, cocaine use disorder; SMD, standardized mean difference.

**TABLE 4:** CUD pathways targeted by ketamine.

No.	Pathways^a^

1	Neuroactive ligand-receptor interaction
2	Alcoholism
3	cAMP signaling pathway
4	Cocaine addiction
5	Dopaminergic synapse
6	Amphetamine addiction
7	GABAergic synapse
8	Morphine addiction
9	Serotonergic synapse
0	Nicotine addiction
11	Retrograde endocannabinoid signaling
12	Long-term depression

Abbreviation: CUD, cocaine use disorder.

a From 78 significantly enriched pathways for ketamine.

## Data Availability

The data that support the findings of this study are openly available in “https://trinetx.com/”.
