# Supplementary material for: Repurposing ketamine to treat cocaine use disorder: integration of artificial intelligence-based prediction, expert evaluation, clinical corroboration and mechanism of action analyses
Source: Addiction. Author manuscript; Available in PMC 2023 Nov 8. (PMC10631254; doi:10.1111/add.16168)
Supplement: Supporting information [file NIHMS1940319-supplement-Supporting_information.docx]

**Supporting Information**

**1. Figure S1.** Comparison of gender and race in remission from CUD among patients taking ketamine as an anesthetic

**2. Figure S2.** Comparison of gender and race in remission from CUD among patients taking ketamine as an antidepressant

**3. Table S1.** Statistics of nodes and interactions in the knowledge graph

**4. Table S2.** Covariates and their standardized names, codes, and data types used in the TriNetX platform

**5. Table S3.** Top 35 drug candidates ranked by our AI-based drug discovery system

**6. Table S4.** Advisory committee ratings of the top-35 drugs

**7. Table S5.** Characteristics of CUD patients prescribed ketamine vs another anesthetic in the anesthesia group

**8. Table S6.** Characteristics of CUD patients prescribed ketamine vs antidepressants/midazolam in the depression group

**9. Table S7.** The complete list of CUD- and ketamine-associated genes

**10. Table S8.** The full list enriched pathways for cocaine use disorder and ketamine

**1. Figure S1.** Comparison of gender and race in remission from CUD among patients taking ketamine as an anesthetic.


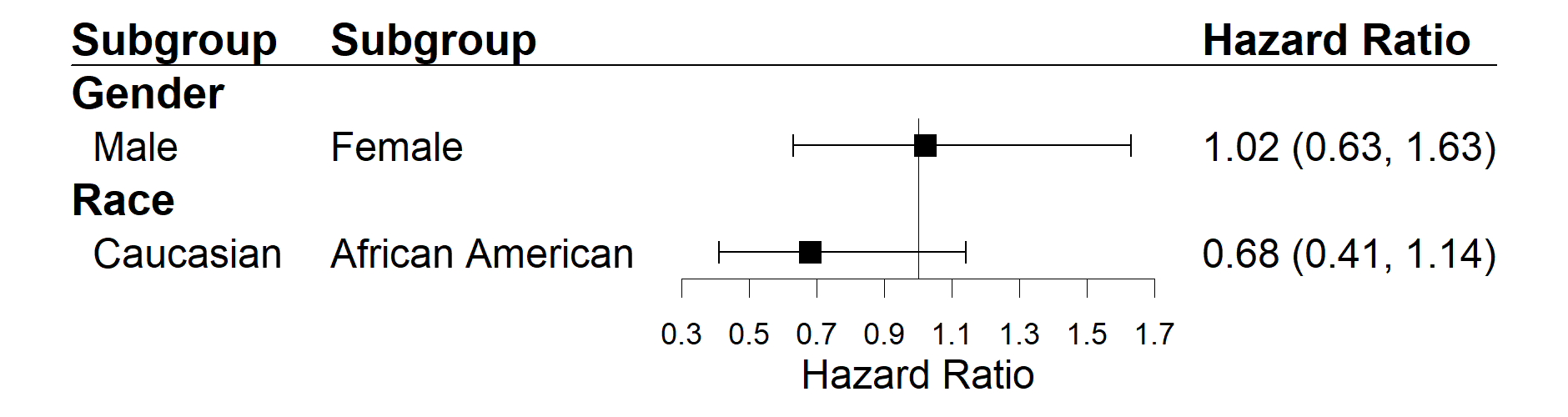


**2. Figure S2.** Comparison of gender and race in remission from CUD among patients taking ketamine as an antidepressant.


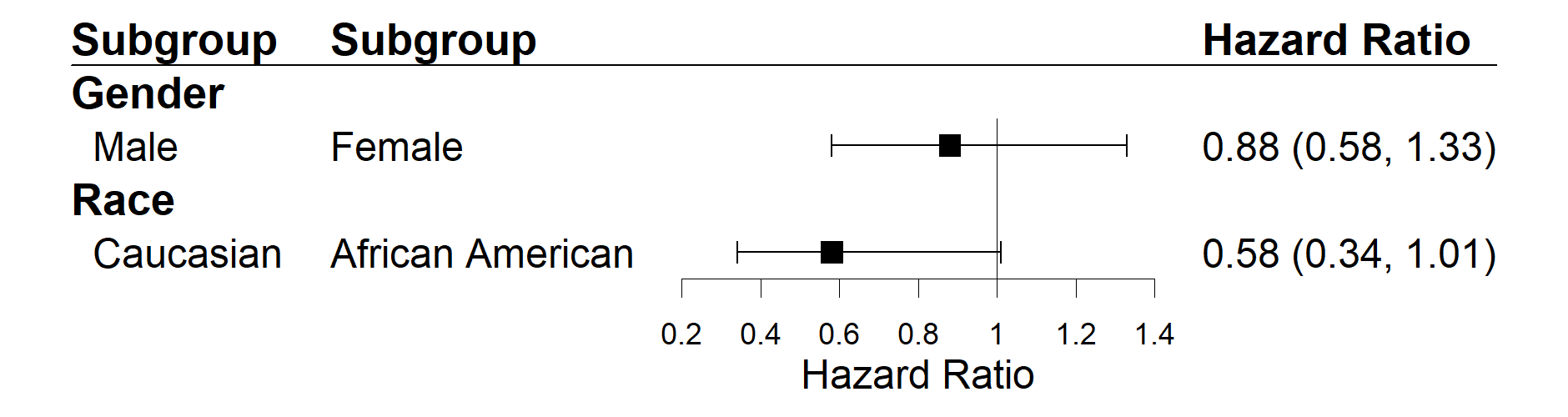


# 3. Table S1. Statistics of nodes and interactions in the knowledge graph

| **Knowledge Type** | **Data Source** | **Interaction**  **Type** | **Interaction**  **Number** | **Node Type** | **Node**  **Number** |
| --- | --- | --- | --- | --- | --- |
| Phenome-level  knowledge | Phenomebrowser  Database | Drug-Mammalian  Phenotype | 36,422 | Drug | 1,228 |
|  |  |  |  | Mammalian  Phenotype | 1,363 |
|  |  | Drug-Human  Phenotype  Ontology | 175,713 | Drug | 1,429 |
|  |  |  |  | Human Phenotype Ontology | 3,003 |
|  | Mouse Genome  Informatics (MGI)  Database | Gene-Mammalian  Phenotype | 187,304 | Gene | 12,219 |
|  |  |  |  | Mammalian  Phenotype | 9,916 |
|  | Gene Ontology  Annotation (GOA)  Database | Gene-Gene  Ontology | 204,862 | Gene | 16,283 |
|  |  |  |  | Gene Ontology | 15,924 |
|  | Genotype-Tissue  Expression (GTEx)  database | Gene-Uberon  Anatomy  Ontology | 539,845 | Gene | 16,579 |
|  |  |  |  | Uberon Anatomy Ontology | 51 |
|  | Human Phenotype  Ontology (HPO)  database | Disease-Human  Phenotype  Ontology | 87,154 | Disease | 7,172 |
|  |  |  |  | Human Phenotype Ontology | 6,784 |
| Genome-level  knowledge | DrugBank database | Drug-Gene | 5,280 | Drug | 985 |
|  |  |  |  | Gene | 1,365 |
|  | Mouse Genome  Informatics (MGI)  Database | Gene-Disease | 7,382 | Gene | 3,363 |
|  |  |  |  | Disease | 4,350 |
| Text-mined  knowledge | TreatKB | Drug-Disease | 2,764 | Drug | 639 |
|  |  |  |  | Disease | 371 |

**4. Table S2.** Covariates and their standardized names, codes, and data types used in the TriNeX database

| **Covariate** | **Name (abbreviation or code)** | **Datatype** |
| --- | --- | --- |
| Age | Age at index (AI) | Continuous |
| Sex | Female (F) | Present/absent |
|  | Male (M) | Present/absent |
| Ethnicity | Hispanic/Latinx (2135-2) | Present/absent |
|  | Not Hispanic/Latinx (2186-5) | Present/absent |
| Race | African American/Black (2054-5) | Present/absent |
|  | White (2106-3) | Present/absent |
|  | Asian (2028-9) | Present/absent |
| Comorbidities | Mental and behavioral disorders due to psychoactive substance use  (F10-F19) | Present/absent |
|  | Mood [affective] disorders (F30-F39) | Present/absent |
|  | Anxiety, dissociative, stress-related, somatoform and other nonpsychotic mental disorders (F40-F48) | Present/absent |
|  | Schizophrenia, schizotypal, delusional, and other non-mood psychotic disorders (F20-F29) | Present/absent |
|  | Hypertensive diseases (I10-I16) | Present/absent |
|  | Ischemic heart diseases (I20-I25) | Present/absent |
|  | Other forms of heart disease (I30-I5A) | Present/absent |
|  | Cerebrovascular diseases (I60-I69) | Present/absent |
|  | Acute kidney failure (N17) | Present/absent |
|  | Antisocial personality disorder (F60.2) | Present/absent |
|  | Conduct disorders (F91) | Present/absent |
|  | Attention-deficit hyperactivity disorders (F90) | Present/absent |
| Socioeconomic and  psychosocial status | Anxiety, dissociative, stress-related, somatoform and other nonpsychotic mental disorders (F40-F48) | Present/absent |

**5. Table S3.** Top 35-ranked drug candidates for the CTN-0114 advisory committee review

| **No.** | **Drug** | **Original Indication** | **Evidence for CUD Treatment** |
| --- | --- | --- | --- |
| 1 | Amoxapine | Depression |  |
| 2 | Aripiprazole | Schizophrenia | NCT00780702, NCT00276874 |
| 3 | Loxapine | Schizophrenia |  |
| 4 | Olanzapine | Schizophrenia |  |
| 5 | Amitriptyline | Depression |  |
| 6 | Ketamine | Anesthetic agent, Depression | NCT01535937, NCT01790490 |
| 7 | Clozapine | Schizophrenia | PMID10891628 |
| 8 | Quetiapine | Schizophrenia | NCT00232336, NCT00631748 |
| 9 | Ziprasidone | Schizophrenia |  |
| 10 | Trimipramine | Depression |  |
| 11 | Dopamine | Hemodynamic imbalances |  |
| 12 | Haloperidol | Schizophrenia |  |
| 13 | Promethazine | Allergic conditions | NCT02037126 |
| 14 | Mianserin | Depression |  |
| 15 | Meperidine | Pain |  |
| 16 | Amphetamine | Attention Deficit Hyperactivity Disorder | NCT00218348, NCT00000308, NCT00218062 |
| 17 | Paroxetine | Vasomotor symptoms |  |
| 18 | Desipramine | Depression | NCT00000271, NCT00000245 |
| 19 | Imipramine | Depression |  |
| 20 | Lamotrigine | Seizures | NCT00280293, NCT00015106 |
| 21 | Bromocriptine | Hyperprolactinemia-Associated Dysfunctions |  |
| 22 | Cabergoline | hyperprolactinemic disorders | NCT01651364, NCT00033111 |
| 23 | Asenapine | Schizophrenia | PMID30858643 |
| 24 | Lisuride | Parkinson's Disease | PMID8054406 |
| 25 | Butabarbital |  |  |
| 26 | Ergotamine | Migraines |  |
| 27 | Pentobarbital | Sedatives |  |
| 28 | Atomoxetine | Attention-Deficit/Hyperactivity Disorder | NCT01802515, NCT00218543 |
| 29 | Chlorpromazine | Schizophrenia |  |
| 30 | Nortriptyline | Depression |  |
| 31 | Enflurane | Anesthetic agent |  |
| 32 | Paliperidone | Schizophrenia |  |
| 33 | Etomidate | Anesthetic agent |  |
| 34 | Isoflurane | Anesthetic agent |  |
| 35 | Acamprosate | Alcohol dependence | NCT00385268 |

NCT: drugs from clinical trials. PMID: drugs from biomedical literature.

**6. Table S4.** Advisory committee ratings of the top-35 drugs

| **Drug** | **Original Indication** | **Likely Poor Adherence/ Problem?***  **No** | **Likely helpful for co-occurring substance use?***  **Yes** | **Likely helpful for phenotypes that are barriers to recovery?***  **Yes** | **Being Investigated for CUD? ***  **No** | **EHR analysis? ***  **Yes** |
| --- | --- | --- | --- | --- | --- | --- |
| Ketamine | Anesthetic agent | 7 (100%) | 7 (100%) | 6 (85.7%) | 3 (42.9%) | 7 (100%) |
| Amphetamine | ADHD | 3 (42.9%) | 2 (28.6%) | 5 (71.4%) | 5 (71.4%) | 6 (85.7%) |
| Cabergoline | Hyperprolactine Mic. Disorders | 6 (85.7%) | 0 (0.00%) | 1 (14.3%) | 7 (100%) | 4 (57.1%) |
| Lisuride | Parkinson's Disease | 4 (57.1%) | 0 (0.00%) | 1 (14.3%) | 7 (100%) | 4 (57.1%) |
| Nortriptyline | Depression | 4 (57.1%) | 2 (28.6%) | 5 (71.4%) | 7 (100%) | 4 (57.1%) |
| Quetiapine | Schizophrenia | 0 (0.00%) | 2 (28.6%) | 4 (57.1%) | 7 (100%) | 4 (57.1%) |
| Ziprasidone | Schizophrenia | 2 (28.6%) | 1 (14.3%) | 4 (57.1%) | 7 (100%) | 4 (57.1%) |
| Acamprosate | Alcohol dependence | 4 (57.1%) | 5 (71.4%) | 1 (14.3%) | 6 (85.7%) | 3 (42.9%) |
| Asenapine | Schizophrenia | 4 (57.1%) | 1 (14.3%) | 3 (42.9%) | 7 (100%) | 3 (42.9%) |
| Atomoxetine | Attention-Deficit/Hyperactivity Disorder | 6 (85.7%) | 3 (42.9%) | 6 (85.7%) | 5 (71.4%) | 3 (42.9%) |
| Desipramine | Depression | 5 (71.4%) | 0 (0.00%) | 4 (57.1%) | 6 (85.7%) | 3 (42.9%) |
| Imipramine | Depression | 4 (57.1%) | 1 (14.3%) | 4 (57.1%) | 7 (100%) | 3 (42.9%) |
| Lamotrigine | Seizures | 3 (42.9%) | 2 (28.6%) | 5 (71.4%) | 6 (85.7%) | 3 (42.9%) |
| Loxapine | Schizophrenia | 1 (14.3%) | 2 (28.6%) | 4 (57.1%) | 7 (100%) | 3 (42.9%) |
| Mianserin | Depression | 3 (42.9%) | 0 (0.00%) | 4 (57.1%) | 7 (100%) | 3 (42.9%) |
| Olanzapine | Schizophrenia | 0 (0.00%) | 2 (28.6%) | 5 (71.4%) | 6 (85.7%) | 3 (42.9%) |
| Paliperidone | Schizophrenia | 3 (42.9%) | 1 (14.3%) | 5 (71.4%) | 7 (100%) | 3 (42.9%) |
| Amoxapine | Depression | 1 (14.3%) | 1 (14.3%) | 4 (57.1%) | 7 (100%) | 2 (28.6%) |
| Bromocriptine | Hyperprolactinemia-Associated Dysfunctions | 5 (71.4%) | 0 (0.00%) | 1 (14.3%) | 7 (100%) | 2 (28.6%) |
| Ergotamine | Migraines | 3 (42.9%) | 0 (0.00%) | 0 (0.00%) | 7 (100%) | 2 (28.6%) |
| Paroxetine | Vasomotor symptoms | 3 (42.9%) | 1 (14.3%) | 4 (57.1%) | 7 (100%) | 2 (28.6%) |
| Promethazine | Allergic conditions | 3 (42.9%) | 0 (0.00%) | 2 (28.6%) | 7 (100%) | 2 (28.6%) |
| Amitriptyline | Depression | 1 (14.3%) | 0 (0.00%) | 4 (57.1%) | 7 (100%) | 1 (14.3%) |
| Aripiprazole | Schizophrenia | 2 (28.6%) | 2 (28.6%) | 3 (42.9%) | 6 (85.7%) | 1 (14.3%) |
| Chlorpromazine | Schizophrenia | 1 (14.3%) | 1 (14.3%) | 3 (42.9%) | 6 (85.7%) | 1 (14.3%) |
| Dopamine | Hemodynamic imbalances | 1 (14.3%) | 3 (42.9%) | 0 (0.00%) | 7 (100%) | 1 (14.3%) |
| Etomidate | Anesthetic agent | 3 (42.9%) | 0 (0.00%) | 0 (0.00%) | 7 (100%) | 1 (14.3%) |
| Haloperidol | Schizophrenia | 0 (0.00%) | 0 (0.00%) | 2 (28.6%) | 7 (100%) | 1 (14.3%) |
| Meperidine | Pain | 2 (28.6%) | 1 (14.3%) | 0 (0.00%) | 7 (100%) | 1 (14.3%) |
| Trimipramine | Depression | 2 (28.6%) | 1 (14.3%) | 2 (28.6%) | 7 (100%) | 1 (14.3%) |
| Butabarbital | Sedative | 2 (28.6%) | 0 (0.00%) | 0 (0.00%) | 7 (100%) | 0 (0.00%) |
| Clozapine | Schizophrenia | 0 (0.00%) | 2 (28.6%) | 5 (71.4%) | 7 (100%) | 0 (0.00%) |
| Enflurane | Anesthetic Agent | 3 (42.9%) | 0 (0.00%) | 0 (0.00%) | 7 (100%) | 0 (0.00%) |
| Isoflurane | Anesthetic agent | 3 (42.9%) | 0 (0.00%) | 0 (0.00%) | 7 (100%) | 0 (0.00%) |
| Pentobarbital | Sedatives | 2 (28.6%) | 0 (0.00%) | 0 (0.00%) | 7 (100%) | 0 (0.00%) |

*Note: a greater % indicates a more favorable profile for a drug candidate across all of the questions in this table.

**7. Table S5.** Characteristics of CUD patients prescribed ketamine vs another anesthetic in the anesthesia group

| **Characteristics** | **Before Matching** | | | **After Matching** | | |
| --- | --- | --- | --- | --- | --- | --- |
|  | **Ketamine Cohort** | **Anesthetic Cohort** | **SMD** | **Ketamine Cohort** | **Anesthetic Cohort** | **SMD** |
| Total No. | 1,515 | 5,833 |  | 1,508 | 1,508 |  |
| Age, mean (SD) | 43.6 (13.7) | 49.2(13.1) | **0.41*** | 43.7(13.7) | 43.2(14) | 0.03 |
| ***Sex, %*** | | | | | | |
| Female | 24.6 | 25.5 | 0.01 | 24.7 | 23.6 | 0.02 |
| Male | 75.1 | 74.2 | 0.02 | 75.1 | 76.2 | 0.02 |
| ***Ethnicity, %*** | | | | | | |
| Hispanic/Latinx | 10.5 | 7.2 | **0.11*** | 10.6 | 9.1 | 0.04 |
| Not Hispanic/  Latinx | 82.1 | 87.1 | **0.13*** | 82.2 | 84.4 | 0.05 |
| ***Race, %*** | | | | | | |
| African American/Black | 38.6 | 50.9 | **0.24*** | 38.8 | 37.6 | 0.02 |
| White | 51.1 | 41.1 | **0.19*** | 50.8 | 52.9 | 0.04 |
| Asian | 0.6 | 0.3 | 0.04 | 0.6 | 0.6 | 0 |
| ***Comorbidities, %*** | | | | | | |
| Mental and behavioral  disorders | 82.5 | 77.8 | **0.11*** | 82.4 | 81.7 | 0.02 |
| Anxiety | 20.5 | 15.6 | **0.12*** | 20.2 | 18.4 | 0.04 |
| Schizophrenia | 4.2 | 3.8 | 0.01 | 4.1 | 3.3 | 0.04 |
| Hypertensive diseases | 36.9 | 48.3 | **0.23*** | 37.1 | 35.4 | 0.03 |
| Ischemic heart diseases | 16.3 | 21.1 | **0.12*** | 16.4 | 14.7 | 0.04 |
| Other forms of heart disease | 38.4 | 38.1 | 0.009 | 38.4 | 36.1 | 0.047 |
| Cerebrovascular diseases | 13.1 | 15.9 | 0.08 | 13.1 | 11.1 | 0.055 |
| Acute kidney failure | 20.2 | 16.7 | 0.08 | 19.8 | 17.3 | 0.06 |
| Antisocial personality  disorder | 0.6 | 0.2 | 0.07 | 0.6 | 0.6 | 0 |
| Conduct disorder | 2.5 | 1.3 | 0.09 | 2.5 | 1.9 | 0.03 |
| ADHD | 2.3 | 1.2 | 0.08 | 2.2 | 1.9 | 0.01 |
| Socioeconomic and  psychosocial status | 13.1 | 12.1 | 0.03 | 13.1 | 11.6 | 0.04 |

SMD = standardized mean difference; ADHD = Attention-deficit/hyperactivity disorder. *Values shown in bold type are significant at SMD > 0.1.

**8. Table S6.** Characteristics of CUD patients prescribed ketamine vs antidepressants/midazolam in the depression group

| **Characteristics** | **Before Matching** | | | **After Matching** | | |
| --- | --- | --- | --- | --- | --- | --- |
|  | **Ketamine Cohort** | **Antidepressant Cohort** | **SMD** | **Ketamine Cohort** | **Antidepressant Cohort** | **SMD** |
| Total No. | 1,785 | 36,030 |  | 1,779 | 1,779 |  |
| Age, mean (SD) | 41.6(13.9) | 41.3(13.7) | 0.01 | 41.5(13.9) | 41.5(13.9) | 0.009 |
| ***Sex, %*** | | | | | | |
| Female | 42.1 | 41.4 | 0.01 | 42.1 | 42.6 | 0.01 |
| Male | 56.1 | 57.57% | 0.02 | 56.1 | 55.6 | 0.01 |
| ***Ethnicity, %*** | | | | | | |
| Hispanic/Latinx | 7.8 | 5.9 | 0.07 | 7.8 | 8.1 | 0.01 |
| Not Hispanic/  Latinx | 68.6 | 74.1 | **0.12*** | 68.7 | 69.2 | 0.01 |
| ***Race, %*** | | | | | | |
| African American/Black | 22.3 | 32.1 | **0.22*** | 22.3 | 22.1 | 0.005 |
| White | 56.2 | 56.1 | 0.001 | 56.3 | 56.1 | 0.003 |
| Asian | 0.8 | 0.6 | 0.02 | 0.8 | 0.8 | 0.006 |
| ***Comorbidities, %*** | | | | | | |
| Mental and behavioral  disorders | 100 | 100 |  | 100 | 100 |  |
| Anxiety | 67.2 | 62.4 | **0.11*** | 67.2 | 66.6 | 0.01 |
| Schizophrenia | 19.9 | 20.7 | 0.02 | 19.8 | 18.1 | 0.04 |
| Hypertensive diseases | 45.1 | 35.1 | **0.21*** | 44.8 | 45.1 | 0.003 |
| Ischemic heart diseases | 15.9 | 10.6 | **0.15*** | 15.9 | 15.5 | 0.01 |
| Other forms of heart disease | 38.3 | 21.4 | **0.37*** | 38.1 | 39.1 | 0.025 |
| Cerebrovascular diseases | 11.1 | 6.9 | **0.14*** | 10.9 | 10.6 | 0.01 |
| Acute kidney failure | 27.2 | 10.7 | **0.42*** | 27.1 | 26.1 | 0.02 |
| Antisocial personality  disorder | 1.5 | 1.6 | 0.009 | 1.5 | 1.4 | 0.009 |
| Conduct disorder | 5.4 | 3.7 | 0.08 | 5.4 | 5.5 | 0.004 |
| ADHD | 9.6 | 8.7 | 0.03 | 9.6 | 9.5 | 0.003 |
| Socioeconomic and  psychosocial status | 28.1 | 28.6 | 0.01 | 28.1 | 27.2 | 0.01 |

SMD = standardized mean difference; ADHD = Attention-deficit/hyperactivity disorder. *Values shown in bold type are significant at SMD > 0.1.

**9. Table S7.** The complete list of CUD- and ketamine-associated genes.

| **Item** | **Total Number** | **List** |
| --- | --- | --- |
| Cocaine Use Disorder | 22 | DRD3, GABRA2, CAMK4, MECP2, OPRK1, COMT, CREB1, CARTPT, CRH, CNR1, CRHR1, OPRM1, SLC6A4, NPY, PDYN, DRD2, HTR1B, SLC6A3, EGR1, GAD1, GABRB3, BDNF |
| Ketamine | 154 | GRIN2A, GRIN2B, GRIN2C, GRIN2D, TMPRSS11D, GRIN3B, GRIN3A, GABRB3, GABRB2, OPRM1, OPRL1, OPRD1, OPRK1, GABRD, CHRNA4, GABRB1, CHRNA2, FOS, CHRNA5, CHRNB4, STXBP5, GABRP, CHRNB2, CHRND, CHRNA1, GABRE, CHRNA3, CHRNB3, OGFR, GABRR1, GABRR2, CHRNE, GABRA1, GABRR3, GRM3, BDNF, IL4, TNF, ALB, NPBWR1, GRM2, ENSRNOG00000048887, HTR2A, GRIN1, GRIK1, CHRNA7, LOC497963, NOS3, GRIK2, HSPA4, GRIA2, GRM5, GRM1, HMOX1, GRIK5, HMGB1, HSP72, HSPA1A, ADNP, CDC42, GRM6, POMC, SCNN1G, TNNT1, SNAI2, CDH1, BCHE, SNAI1, SLC6A4, CSN3, HTR2C, CHRNG, OGFRL1, GRIK4, ENSRNOG00000046581, GSK3B, MAPK14, CYP2B1, GRM4, CYP19A1, ENSRNOG00000050823, CYP2B15, MAPK11, GLUT3, SLC2A3, CYP3A1, GABRA2, GABRA3, GABRA5, GABRG2, CDH3, AFP, GRIA1, GRID2, GRID1, HMOX2, ENSRNOG00000032560, CYP3A18, GABRG1, AFM, SLC6A3, GABRA6, GABRA4, HTR3A, ACHE, SLC2A4, CXCR4, DRD2, MAPK3, GABRQ, SLC2A2, CES2F, GLRA4, GLRA2, MAPK12, MAPK13, VEGFA, GAD1, ADORA2A, SLC2A1, GRIK3, AGTR1A, SLC2A5, CES1A, RHO, TBXAS1, CYP3A62, TNNT3, TNNT2, HTR1A, OPN4, ENSRNOG00000050802, GABRG3, CYP3A2, SSTR1, CCR5, HRH3, GPR85, NPY1R, GALR2, GPR22, CNR1, GPR37L1, MAPK7, GRM8, SSTR5, CASR, GRIA3, GRIA4, MAPK1, APLNR, AGTR2, PVALB, AGTR1B |

**10. Table S8.** The full list enriched pathways for cocaine use disorder and ketamine

| **Item** | **Total Number** | **Pathways** | **Pathway ID** |
| --- | --- | --- | --- |
| Cocaine Use  Disorder | 13 | Neuroactive ligand-receptor interaction | hsa04080 |
|  |  | Alcoholism | hsa05034 |
|  |  | cAMP signaling pathway | hsa04024 |
|  |  | Cocaine addiction | hsa05030 |
|  |  | Dopaminergic synapse | hsa04728 |
|  |  | Amphetamine addiction | hsa05031 |
|  |  | GABAergic synapse | hsa04727 |
|  |  | Morphine addiction | hsa05032 |
|  |  | Serotonergic synapse | hsa04726 |
|  |  | Nicotine addiction | hsa05033 |
|  |  | Retrograde endocannabinoid signaling | hsa04723 |
|  |  | Cushing syndrome | hsa04934 |
|  |  | Long-term depression | hsa04730 |
| Ketamine | 78 | Neuroactive ligand-receptor interaction | hsa04080 |
|  |  | Nicotine addiction | hsa05033 |
|  |  | Retrograde endocannabinoid signaling | hsa04723 |
|  |  | Glutamatergic synapse | hsa04724 |
|  |  | GABAergic synapse | hsa04727 |
|  |  | Morphine addiction | hsa05032 |
|  |  | Cocaine addiction | hsa05030 |
|  |  | cAMP signaling pathway | hsa04024 |
|  |  | Amphetamine addiction | hsa05031 |
|  |  | Long-term potentiation | hsa04720 |
|  |  | Dopaminergic synapse | hsa04728 |
|  |  | Circadian entrainment | hsa04713 |
|  |  | Taste transduction | hsa04742 |
|  |  | IL-17 signaling pathway | hsa04657 |
|  |  | VEGF signaling pathway | hsa04370 |
|  |  | T cell receptor signaling pathway | hsa04660 |
|  |  | Rap1 signaling pathway | hsa04015 |
|  |  | Prolactin signaling pathway | hsa04917 |
|  |  | Pathways of neurodegeneration - multiple diseases | hsa05022 |
|  |  | Leishmaniasis | hsa05140 |
|  |  | AGE-RAGE signaling pathway in diabetic complications | hsa04933 |
|  |  | Prion disease | hsa05020 |
|  |  | Alcoholism | hsa05034 |
|  |  | Fc epsilon RI signaling pathway | hsa04664 |
|  |  | Serotonergic synapse | hsa04726 |
|  |  | Spinocerebellar ataxia | hsa05017 |
|  |  | Neurotrophin signaling pathway | hsa04722 |
|  |  | Growth hormone synthesis; secretion and action | hsa04935 |
|  |  | Pertussis | hsa05133 |
|  |  | Lipid and atherosclerosis | hsa05417 |
|  |  | Long-term depression | hsa04730 |
|  |  | Yersinia infection | hsa05135 |
|  |  | Fluid shear stress and atherosclerosis | hsa05418 |
|  |  | Toxoplasmosis | hsa05145 |
|  |  | Cholinergic synapse | hsa04725 |
|  |  | Gap junction | hsa04540 |
|  |  | Calcium signaling pathway | hsa04020 |
|  |  | Th1 and Th2 cell differentiation | hsa04658 |
|  |  | Sphingolipid signaling pathway | hsa04071 |
|  |  | GnRH signaling pathway | hsa04912 |
|  |  | Relaxin signaling pathway | hsa04926 |
|  |  | Chagas disease | hsa05142 |
|  |  | Toll-like receptor signaling pathway | hsa04620 |
|  |  | Th17 cell differentiation | hsa04659 |
|  |  | TNF signaling pathway | hsa04668 |
|  |  | PD-L1 expression and PD-1 checkpoint pathway in cancer | hsa05235 |
|  |  | Phospholipase D signaling pathway | hsa04072 |
|  |  | MAPK signaling pathway | hsa04010 |
|  |  | Platelet activation | hsa04611 |
|  |  | Human cytomegalovirus infection | hsa05163 |
|  |  | Type II diabetes mellitus | hsa04930 |
|  |  | Kaposi sarcoma-associated herpesvirus infection | hsa05167 |
|  |  | Endocrine resistance | hsa01522 |
|  |  | Osteoclast differentiation | hsa04380 |
|  |  | Adherens junction | hsa04520 |
|  |  | FoxO signaling pathway | hsa04068 |
|  |  | C-type lectin receptor signaling pathway | hsa04625 |
|  |  | Estrogen signaling pathway | hsa04915 |
|  |  | Human immunodeficiency virus 1 infection | hsa05170 |
|  |  | Adrenergic signaling in cardiomyocytes | hsa04261 |
|  |  | Non-alcoholic fatty liver disease | hsa04932 |
|  |  | Pathogenic Escherichia coli infection | hsa05130 |
|  |  | Hepatitis B | hsa05161 |
|  |  | Bladder cancer | hsa05219 |
|  |  | Inflammatory mediator regulation of TRP channels | hsa04750 |
|  |  | Proteoglycans in cancer | hsa05205 |
|  |  | Renal cell carcinoma | hsa05211 |
|  |  | RIG-I-like receptor signaling pathway | hsa04622 |
|  |  | Epithelial cell signaling in Helicobacter pylori infection | hsa05120 |
|  |  | Progesterone-mediated oocyte maturation | hsa04914 |
|  |  | Chemical carcinogenesis - receptor activation | hsa05207 |
|  |  | Insulin resistance | hsa04931 |
|  |  | Signaling pathways regulating pluripotency of stem cells | hsa04550 |
|  |  | HIF-1 signaling pathway | hsa04066 |
|  |  | Chemical carcinogenesis - reactive oxygen species | hsa05208 |
|  |  | NOD-like receptor signaling pathway | hsa04621 |
|  |  | Leukocyte transendothelial migration | hsa04670 |
|  |  | Amyotrophic lateral sclerosis | hsa05014 |
